# Biogeographic Patterns of Structural Traits and C:N:P Stoichiometry of Tree Twigs in China’s Forests

**DOI:** 10.1371/journal.pone.0116391

**Published:** 2015-02-09

**Authors:** Fanyun Yao, Yahan Chen, Zhengbing Yan, Peng Li, Wenxuan Han, Jingyun Fang

**Affiliations:** 1 Key Lab for Earth Surface Processes of the Ministry of Education, Department of Ecology, College of Urban and Environmental Sciences, Peking University, Beijing, China; 2 College of Resources and Environmental Sciences, China Agricultural University, Beijing, China; Fudan University, CHINA

## Abstract

There have been a number of studies on biogeographic patterns of plant leaf functional traits; however, the variations in traits of other plant organs such as twigs are rarely investigated. In this study, we sampled current-year twigs of 335 tree species from 12 forest sites across a latitudinal span of 32 degrees in China, and measured twig specific density (TSD), twig dry matter content (TDMC), and carbon (C), nitrogen (N) and phosphorous (P) contents, to explore the latitudinal and environmental patterns of these twig traits. The overall mean of TSD and TDMC was 0.37 g cm^−3^ and 41%, respectively; mean twig C, N and P was 472 mg g^−1^, 9.8 mg g^−1^ and 1.15 mg g^−1^, respectively, and mean N:P mass ratio was 10.6. TSD was positively correlated with TDMC which was positively associated with twig C but negatively with twig N and P. There were no significant differences in TSD between conifer, deciduous-broadleaf and evergreen-broadleaf plants, but evergreen-broadleaf plants had the lowest and conifers the highest TDMC. Conifer twigs were lowest in C, N, P and N:P, whereas deciduous-plant twigs were highest in N and P and evergreen-plant twigs were highest in C and N:P. As latitude increased or temperature/precipitation dropped, TDMC and P increased, but N:P ratio decreased. Our results also showed that the patterns of twig P and N:P stoichiometry were consistent with those reported for leaves, but no significant trends in twig N were observed along the gradient of latitude, climate and soils. This study provides the first large-scale patterns of the twig traits and will improve our understanding of the biogeochemistry of carbon and other key nutrients in forest ecosystems.

## Introduction

As an independent building block and one of the most active compartments of the whole plant [1,2], twigs of trees provide mechanical support and transport water and nutrients. Resource allocation to twigs is therefore an important aspect of plant life-history strategy [3,4]. These resources include carbon (C), nitrogen (N) and phosphorous (P) as vital elements of all living organisms [5]. Previous studies showed that twigs (twigs defined here as their shoot axes without leaves or reproductive structures) nutrient status may allow better prediction of plant resource state and potential deficiencies in N or P than leaves [6,7]. In addition to nutrient contents, twig dry matter content (TDMC) and twig specific density (TSD) are the other two important functional traits, with the former closely related to plant flammability and the latter reflecting the trade-off between plant growth rate and defensive ability [8].

Correlations between functional traits are often considered as a trade-off of plant life-history strategies or the result of biochemical or structural constraints [9,10]. TDMC is expected to be positively linked with TSD [8], and a high TDMC is usually accompanied by a low N content [11]. Variation in plant functional traits reflects how plants are adapted to the environment [12]. For example, previous studies research showed that leaf functional traits are related to key environmental factors in typical ways [13,14]: leaf N and P change across latitudes and are significantly associated with mean annual temperature (MAT), annual precipitation (AP) and soil nutrients [13,15–17]. Moreover, recent study has revealed that stem, root, and older leaf N:P ratios responded more sensitively to nutrient changes than new foliage [18]. However, variations in twig structural and stoichiometric traits have rarely been investigated, despite their importance for plant performance. Understanding how these twig traits respond to large-scale environmental gradients may help to determine plant strategies and nutrients remobilization [19].

Using data of the structural traits (TSD and TDMC) and stoichiometry (C, N, and P) of the current-year twigs of 335 tree species from 12 forest sites in eastern China [15], we address the following issues: (1) the variation in structural traits and C, N, P stoichiometry of twigs, and their relationships across different plant growth forms; and (2) latitudinal patterns of these twig functional traits, and their responses to environmental gradients.

## Material and Methods

### Ethics Statement

Peking University has had a permit from local forestry authorities (Genhe forestry bureau (http://www.ghlyj.com/), Qingdao forestry bureau (http://ly.qingdao.gov.cn/), Ningbo forestry bureau (http://linyj.ningbo.gov.cn/) and Zhaoqing forestry bureau (http://www.zqlinye.gov.cn/), et al.) to conduct the experiments in each location. This research was carried out in compliance with the laws of People’s Republic of China. The field studies did not involve endangered or protected species.

### Site description

The 12 forest sites in eastern China (S1 Fig.) spanned a range of 32° in latitude from 18.7°N to 50.9°N. MAT and AP range from −5.7°C to 25.3°C and from 423 mm to 2031 mm, respectively. Soil and vegetation types varied greatly across these sites (for details, see S1 Table). All the climate data (including MAT and AP) are from local research literatures or local weather stations.

### Sampling and measurement

Twigs were sampled during the growing season from 2005–2007 according to sampling protocols provided by Cornelissen et al [8]. We first selected 3–5 mature healthy individuals for each species on which the terminal 10–20 cm of the shoot axis was removed. And 5 twigs were sampled for each plant. In total, we sampled 335 species (including 11 coniferous, 134 deciduous-broadleaf, and 190 evergreen-broadleaf trees) belonging to 198 genera and 73 families.

Twig volume was measured by water displacement. Twigs were immersed in water filled cylinders with tweezers and the change in volume recorded. To reduce the error, measuring small twigs with a small cylinder and big twigs with a big cylinder is advised. Through this method, all the stem segments contain air that would be released by water pressure. And afterwards the water-saturated fresh mass (W_fresh_) was determined. All samples were dried for 72 h at 60°C before the determination of dry mass (W_dry_). Then samples were ground using a ball mill (NM200, Retsch, Haan, Germany) to measure element contents. TDMC was calculated as the ratio of W_dry_ to W_fresh_ [8], and TSD as the ratio of W_dry_ to volume. C and N content were measured by an elemental analyzer (2400 II CHNS/O Elemental Analyzer, Perkin-Elmer, USA). P was measured using a molybdate/ascorbic acid method [20].

Three soil samples (A horizon) were collected randomly across the plot at each forest site, and then were mixed thoroughly and air-dried. They were used for determination of soil total N and P. The method for measuring soil total N and P was the same to twig as described above.

### Data analysis

First, we showed our data with summary statistics including sample size, mean and coefficient of variation (CV) for each life form. Then we used t-tests with Bonferroni corrections to examine variation in the measurements among conifer and deciduous- and evergreen-broadleaf species. We used reduced major axis regression (RMA) to explore the relationship between structural traits and C:N:P stoichiometry. Since TDMC and TSD showed a close exponential relationship, we only regressed TDMC against C:N:P stoichiometry. Linear regression was used to explore the latitudinal pattern of functional traits and the relationship between functional traits and environmental factors (MAT, MAP, soil N, soil P) for conifers, deciduous and evergreen species separately.

All analyses were conducted both at individual and site level (the site level refers to the site-means of the different sampled species). In all analyses, data of structural traits were log10-transformed to normalize residual distributions. Because individual- and site-level analyses generated very similar results, we chose the latter to facilitate comparison with previous studies lacking replicated individual data within species [15,17,21]. All statistical analyses were conducted using R.3.0.1 (R Development Core Team 2013).

## Results

### Twig traits and their relationships with different growth forms

For all species pooled together, TSD and TDMC averaged 0.37 g cm^−3^ and 41%, respectively. The mean concentrations of twig C, N and P were 472, 9.78 and 1.15 mg g^−1^, and the mean N:P mass ratio was 10.6 (Table 1). A significant difference in twig C was found among coniferous, deciduous and evergreen species. Conifers had highest twig C but lowest twig N. Evergreen species had the lowest TDMC, but the highest twig N and N:P ratio. Deciduous species had the highest twig P concentration. TSD showed no significant discrepancy among coniferous, evergreen and deciduous species. Twig N and N:P differed significantly between legumes and non-legumes, but TSD, TDMC, C and P did not show a significant difference between the two plant groups.

**Table 1:** Statistics for twig specific density (TSD), dry matter content (TDMC), C, N, P and N:P of woody plants from 12 forest sites in eastern China.

**Life form**	**TSD (g cm^−3^)**			**TDMC (%)**			**C (mg g^−1^)**			**N (mg g^−1^)**			**P (mg g^−1^)**			**N:P**		
	**n**	**Mean**	**CV**	**n**	**Mean**	**CV**	**n**	**Mean**	**CV**	**n**	**Mean**	**CV**	**n**	**Mean**	**CV**	**n**	**Mean**	**CV**
**Conifer**	6	0.32^a^	0.26	19	0.46^a^	0.23	19	501^a^	0.04	19	7.63^b^	0.26	19	1.02^b^	0.51	19	8.2^b^	0.24
**Deciduous**	145	0.37^a^	0.27	282	0.42^a^	0.22	284	474^b^	0.05	284	9.84^a^	0.32	283	1.20^a^	0.40	283	9.4^b^	0.41
**Evergreen**	103	0.37^a^	0.30	149	0.39^b^	0.25	149	464^c^	0.06	149	9.93^a^	0.44	149	1.08^b^	0.68	148	13.2^a^	0.70
**Legume**	21	0.35^a^	0.20	26	0.39^a^	0.19	27	465^a^	0.04	27	13.9^a^	0.32	27	1.06^a^	0.30	27	14.9^a^	0.44
**Non-legume**	233	0.37^a^	0.30	424	0.41^a^	0.24	425	472^a^	0.06	425	9.5^b^	0.36	423	1.16^a^	0.51	423	10.3^b^	0.61
**All**	254	0.37	0.29	450	0.41	0.23	452	472	0.06	452	9.78	0.37	450	1.15	0.50	450	10.6	0.60

Difference in the plant growth forms was tested using t-test with Bonferroni corrections. Superscript letters(a, b and c) indicate significant differences in variables (*p*<0.05).n, samples size from database averaged by species within the same site.

**Figure 1:**
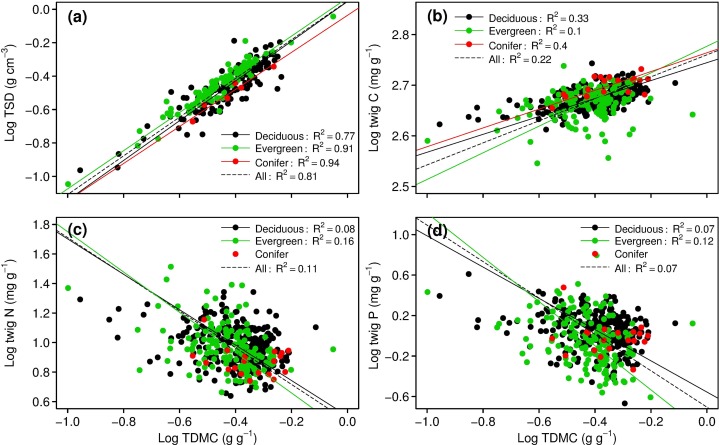
Relationships between twig dry matter content (TDMC) and twig specific density (TSD) and C, N, and P content for different growth forms. (a) TDMC—TSD relationship, (b) TDMC—C relationship, (c) TDMC—N relationship, and (d) TDMC—P relationship. The reduced major axis (RMA) regression is used to fit the relationships.

Fig. 1 illustrated the relationships between twig traits and N and P concentrations. There was strong positive correlation between TSD and TDMC at species level (p<0.001). Conifers, evergreen and deciduous plants shared the same allometric exponent (1.15) (Fig. 1A). There were also significant positive relationships between twig TDMC and twig C for all species pooled and for each growth form (conifer, evergreen and deciduous species) (p<0.05) (Fig. 1B). While significant negative relationships were between TDMC and twig N and P concentration (p<0.05) for deciduous and evergreen species, no significant trend was found for conifers (p = 0.239 and 0.422, respectively) (Fig. 1C and 1D) (For more details, see S2 Table).

### Changes in TDMC and N and P stoichiometry along latitudinal and environmental gradients

**Figure 2:**
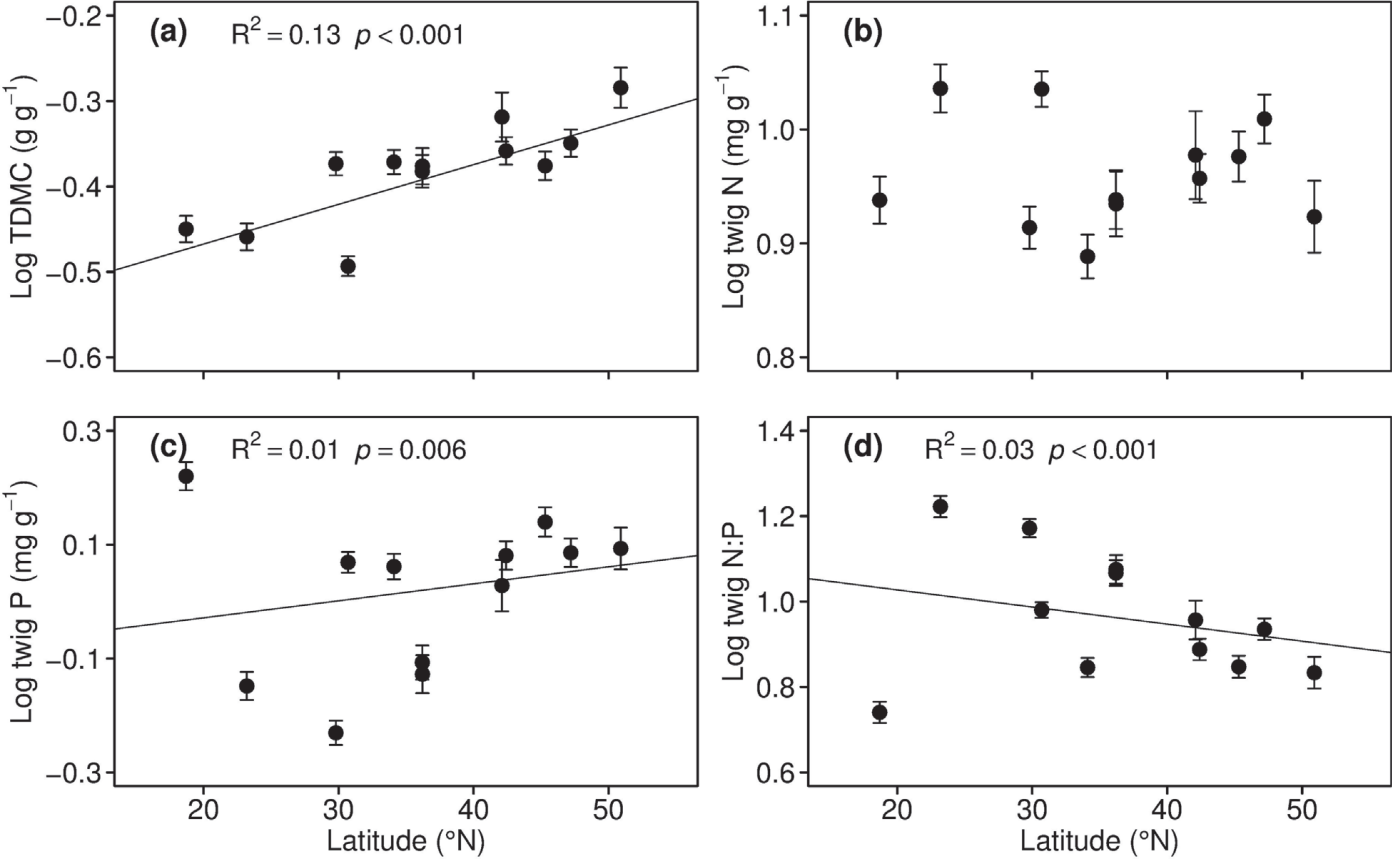
Changes in site-averaged dry matter content (TDMC) and C, N, and P content of twigs with latitude. (a) TDMC, (b) N content, (c) P content, and (d) N:P ratio. Regression lines are fit to the raw data with *p*<0.05. Points and error bars stand for the means and standard errors of the twig traits.

Because TSD showed a close exponential relationship with TDMC, as present before, we only reported the biogeographic patterns of the TDMC and C:N:P stoichiometry. With an increase in latitude, TDMC increased significantly (r^2^ = 0.13, p<0.001; Fig. 2A), and N concentration did not show a clear latitudinal trend (Fig. 2B). The twig P also increased significantly (r^2^ = 0.01, p = 0.006; Fig. 2C), whereas the N:P ratio tended to drop when latitude increased (r^2^ = 0.03, p<0.001; Fig. 2D).

**Figure 3:**
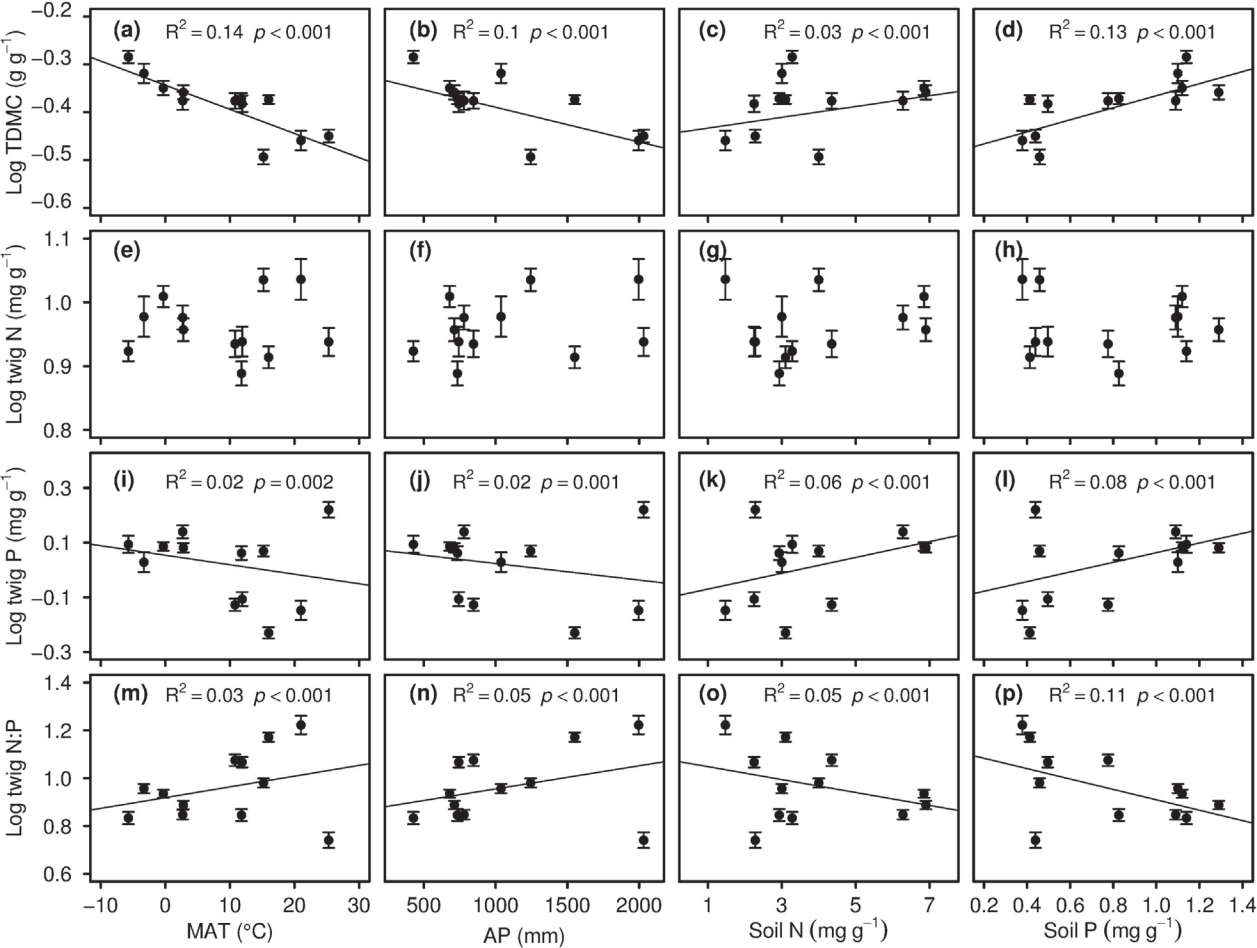
Changes in site-averaged twig TDMC, N, P and N: P along mean annual temperature (MAT), annual precipitation (AP), soil N and soil P. (a-d) twig TDMC; (e-h) twig N; (i-l) twig P; (m-p) twig N:P. Regression lines are fit to the raw data with p<0.05. Points and error bars stand for the means and standard errors of the twig traits.

Fig. 3 presented the changes in the species-pooled mean TDMC and C:N:P stoichiometry with climate (MAT and AP) and soil N and P. As MAT and AP increased, the TDMC and P decreased (with both p<0.001 for TDMC with MAT and AP; p = 0.002 and 0.01 for twig P; Fig. 3A, 3B, 3I, and 3J), while twig N:P ratio increased (with both p<0.001; Fig. 3M and 3N). Along a gradient of soil N and P, the TDMC and C:N:P stoichiometry showed an inverse pattern to those along the climatic gradient. That is, with an increase in soil N and P, TDMC and P increased (with all p<0.001; Fig. 3C, 3D, 3K, and 3L), while twig N:P ratio declined (with both p<0.001; Fig. 3O and 3P). In addition, the twig N did not exhibit a significant pattern along both gradients of climate and soil (Fig. 3E, 3F, 3G, and 3H).

## Discussion

### Comparison of twig C, N, and P contents by growth forms and between twigs and leaves

In this study, we documented the variation in structural traits and C, N, and P stoichiometry of twigs for 335 woody species and provided the first large-scale patterns of twig traits along a gradient of climate and soils across eastern China. We found that neither TSD nor TDMC varied significantly among conifers, evergreen and deciduous trees, while twig C, N, and P concentrations differed significantly among these growth forms, with the highest C content in conifers, N content in evergreen trees, and P in deciduous trees (Table 1).

The different twig C, N, and P stoichiometry by growth form was consistent with that in foliage [22–24], which might be related with the difference in habitats, leaf life span and nutrient resorption [22,25,26]. Compared with leaf stoichiometry reported by our previous study [15], N and P content and N: P ratio in twigs exhibited a much lower value (9.78 vs. 23.2, 1.15 vs. 1.59, and 10.6 vs. 17.6 for twigs verse leave, respectively). These lower values may be linked to the physiological functions of twigs [27–29]. Twigs mainly play a role in supporting and transporting, which involves much fewer metabolic activities (e.g. organic synthesis, photosynthesis, and transpiration) than leaves, which may contribute a lower N and P content in twigs. It was reported that the low twig N contents indicate less N allocated to the non-photosynthetic tissues and more N to foliage for maximizing C assimilation [30,31].

### Patterns of twig N and P stoichiometry along gradients of climate and soil

In this study, the twig P and N:P showed remarkable biogrageophic patterns along the gradient of latitude, climate and soils, but such patterns did not show for the twig N (Fig. 2 and 3). The substantial twig P and N:P patterns are consistent with those of leaves [15,17,32–34]. The tissue P content is directly related to the soil nutrient supply [5]. Because the rain and heat occur in the same season (summer) in eastern China, soil N and P leaching increases with the precipitation increase, leading a reduction of available N and P concentrations in soils in warm and rainy regions [35,36]. This may cause the negative relationship between twig P and MAT and AP. The positive correlation between the twig P and N: P and soil N and P may be because root absorption is the major source of twig N and P. And this result indicates that the N:P ratio of twigs can be used as a responsive indicator of soil nutrient availability. Our study shows that P is more variable than N in twig, suggesting that the twig N:P ratio is mainly determined by the P content.

However, the N trends in the twigs do not coincide with foliar ones reported in our previous study [15], in which we showed that leaf N was significantly associated with latitude and environmental variables. First of all, as a primarily structural organ, twigs have a much lower metabolic active rate (e.g. growth, photosynthesis, respiration) than leaf, which resulting in a lower demand for N (main element of protein). To the non-metabolic organs, the impact of environmental factors on the N content may not be as strong as that in leaf [37]. Secondly, the adequate N supply in atmosphere and multiple mechanisms of N-fixation for plants may also weaken the controlling of climate factors to plant tissues N content [22]. Besides these environmental factors, the allocation strategies of nutrients between different organs and phylogenetic effects may also contribute to the inconsistent pattern of N concentration between twig and leaf along with the shifts in climate [29,38], thus further studies are needed to examine the effects of these variables on the N content in plant twig. Moreover, we found that twig N is more invariable along with latitude and environmental gradients than twig P. Previous studies also suggested that at species level, the homeostatic regulation coefficient of leaf N was higher than that of leaf P, and leaf N was more stable relative to external environment [5,39]. Our findings indicated that the different homeostasis between N and P might also exist in non-leaf organs such as twigs.

Our results show that the relationships between twigs N and P stoichiometry and environment factors are not entirely consistent with those in leaves. This may indicate that the metabolism functions of plant twigs and foliage are not completely coupled [40]. Our findings also present the first large-scale patterns of twig traits and fill the blank in large scale twig element stoichiometry.

## Supporting Information

S1 Fig Locations of sampling sites, spanning 32 degrees in latitude across forests of eastern China. (DOC)

S1 Table Site information. Lat: latitude, Lon: Longitude, MAT: mean annual temperature, and AP: annual precipitation.
(DOC)

S2 Table Relationships between twig TDMC, TSD, C, N, and P. (DOC)

S1 Dataset All traits data based on individual-level. (CSV)

S2 Dataset All traits data based on species-level. (CSV)

S3 Dataset All traits data based on species by site level. (CSV)
