# Supplementary material for: Biogeographic Patterns of Structural Traits and C:N:P Stoichiometry of Tree Twigs in China’s Forests
Source: PLoS One. 2015 Feb 9;10(2):e0116391. doi: 10.1371/journal.pone.0116391 (PMC4321987; doi:10.1371/journal.pone.0116391)
Supplement: S1 Fig — (DOC) [file pone.0116391.s001.doc]

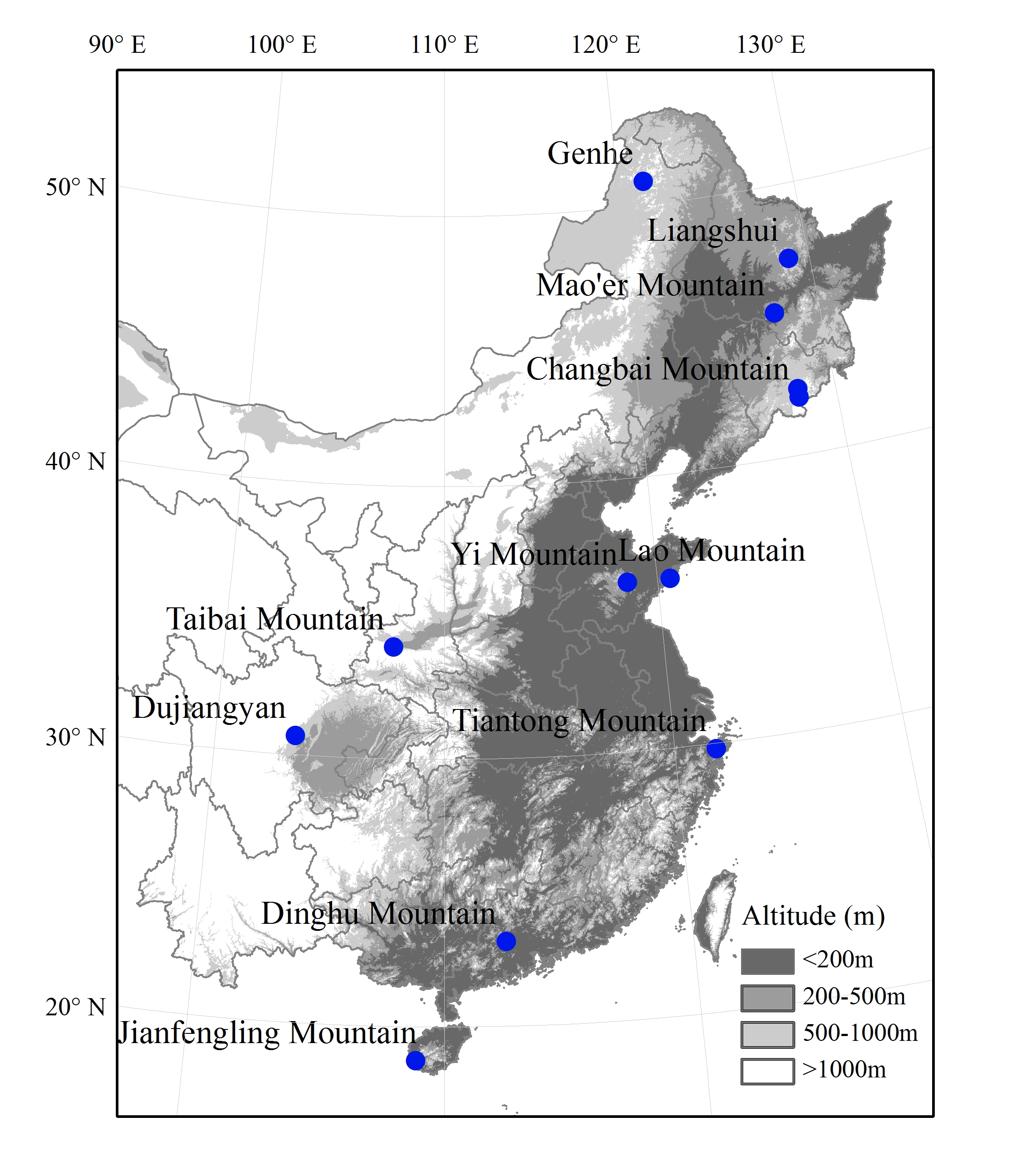


**Figure S1. Locations of sampling sites, spanning 32 degrees in latitude across forests of eastern China**
