# Supplementary material for: Biogeographic Patterns of Structural Traits and C:N:P Stoichiometry of Tree Twigs in China’s Forests
Source: PLoS One. 2015 Feb 9;10(2):e0116391. doi: 10.1371/journal.pone.0116391 (PMC4321987; doi:10.1371/journal.pone.0116391)
Supplement: S1 Table — Lat: latitude, Lon: Longitude, MAT: mean annual temperature, and AP: annual precipitation. (DOC) [file pone.0116391.s002.doc]

**Table S1. Site information. Lat: latitude, Lon: Longitude, MAT: mean annual temperature, and AP: annual precipitation.**

| **Site** | **Lat**  **(°N)** | **Lon**  **(°E)** | **Vegetation type** | **MAT**  **(℃)** | **AP**  **(mm)** | **Soil N**  **(mg g^-1^)** | **Soil P**  **(mg g^-1^)** | **No. of**  **species** |
| --- | --- | --- | --- | --- | --- | --- | --- | --- |
| **Jianfengling** | 18.7 | 108.8 | Tropical seasonal semi-deciduous forest | 25.3 | 2031 | 2.28 | 0.439 | 42 |
| **Dinghu Mountain** | 23.2 | 112.5 | Monsoon evergreen broad-leaved forest | 21 | 1996 | 1.47 | 0.378 | 40 |
| **Tiantong Mountain** | 29.8 | 121.8 | Subtropical evergreen broad-leaved forest | 16 | 1551 | 3.1 | 0.413 | 53 |
| **Dujiangyan** | 30.7 | 103.5 | Subtropical evergreen broad-leaved forest | 15.2 | 1244 | 4 | 0.458 | 74 |
| **Taibai Mountain** | 34.1 | 107.7 | Warm temperate deciduous broad-leaved forest | 11.8 | 734 | 2.93 | 0.826 | 49 |
| **Lao Mountain** | 36.2 | 120.6 | Warm temperate deciduous broad-leaved forest | 11.9 | 743 | 2.25 | 0.496 | 27 |
| **Yi Mountain** | 36.2 | 118.6 | Warm temperate deciduous broad-leaved forest | 10.8 | 846 | 4.35 | 0.776 | 22 |
| **Changbai Mountain 1** | 42.1 | 128.1 | Subalpine Betula ermanii forest | -3.3 | 1038 | 3 | 1.1 | 12 |
| **ChangbaiMountain 2** | 42.4 | 128.1 | Broad-leaved Korean pine forest | 2.8 | 713 | 6.9 | 1.29 | 40 |
| **Mao’er Mountain** | 45.3 | 127.6 | Secondary deciduous broad-leaved forest | 2.7 | 780 | 6.28 | 1.09 | 37 |
| **Liangshui** | 47.2 | 128.9 | Broad-leaved Korean pine forest | -0.3 | 680 | 6.85 | 1.12 | 39 |
| **Genhe** | 50.9 | 121.5 | Temperate coniferous forest | -5.7 | 427 | 3.28 | 1.14 | 19 |
