# Supplementary material for: Biogeographic Patterns of Structural Traits and C:N:P Stoichiometry of Tree Twigs in China’s Forests
Source: PLoS One. 2015 Feb 9;10(2):e0116391. doi: 10.1371/journal.pone.0116391 (PMC4321987; doi:10.1371/journal.pone.0116391)
Supplement: S2 Table — (DOC) [file pone.0116391.s003.doc]

**Table S2. Relationships between twig TDMC, TSD, C, N, and P.**

| **Growth form** | **Individual level** | | | | |  | **Species by Site level** | | | | |
| --- | --- | --- | --- | --- | --- | --- | --- | --- | --- | --- | --- |
|  | ***n*** | ***slope*** | ***s.*CI** | ***r^2^*** | ***p*** |  | ***n*** | ***slope*** | ***s.*CI** | ***r^2^*** | ***p*** |
| **TSD vs. TDMC** | | | | | | | | | | | |
| All | 680 | 1.22 | 1.17,1.28 | 0.68 | <0.001 |  | 254 | 1.16 | 1.10,1.22 | 0.81 | <0.001 |
| Deciduous | 415 | 1.3 | 1.22,1.37 | 0.65 | <0.001 |  | 145 | 1.19 | 1.10,1.29 | 0.77 | <0.001 |
| Evergreen | 244 | 1.16 | 1.10,1.22 | 0.81 | <0.001 |  | 103 | 1.14 | 1.08,1.21 | 0.91 | <0.001 |
| Conifer | 21 | 1.15 | 0.97,1.37 | 0.87 | <0.001 |  | 6 | 1.11 | 0.79,1.55 | 0.94 | 0.001 |
| **C vs. TDMC** | | | | | | | | | | | |
| All | 1533 | 0.24 | 0.23,0.25 | 0.11 | <0.001 |  | 450 | 0.22 | 0.20,0.24 | 0.22 | <0.001 |
| Deciduous | 1021 | 0.18 | 0.17,0.19 | 0.16 | <0.001 |  | 282 | 0.18 | 0.16,0.19 | 0.33 | <0.001 |
| Evergreen | 433 | 0.32 | 0.29,0.35 | 0.04 | <0.001 |  | 149 | 0.26 | 0.23,0.31 | 0.10 | <0.001 |
| Conifer | 79 | 0.28 | 0.23,0.35 | 0.14 | <0.001 |  | 19 | 0.19 | 0.13,0.27 | 0.40 | 0.004 |
| **N vs. TDMC** | | | | | | | | | | | |
| All | 1532 | -1.22 | -1.28,-1.16 | 0.07 | <0.001 |  | 450 | -1.24 | -1.36,-1.14 | 0.11 | <0.001 |
| Deciduous | 1019 | -1.17 | -1.24,-1.10 | 0.04 | <0.001 |  | 282 | -1.20 | -1.34,-1.07 | 0.08 | <0.001 |
| Evergreen | 434 | -1.35 | -1.48,-1.24 | 0.13 | <0.001 |  | 149 | -1.37 | -1.58,-1.18 | 0.16 | <0.001 |
| Conifer | 79 | -1.00 | -1.25,-0.80 | 0.03 | 0.106 |  | 19 | -0.92 | -1.47,-0.57 | 0.08 | 0.239 |
| **P vs. TDMC** | | | | | | | | | | | |
| All | 1531 | -1.89 | -1.99,-1.80 | 0.06 | <0.001 |  | 448 | -1.80 | -1.97,-1.64 | 0.07 | <0.001 |
| Deciduous | 1017 | -1.63 | -1.73,-1.53 | 0.07 | <0.001 |  | 281 | -1.54 | -1.73,-1.38 | 0.07 | <0.001 |
| Evergreen | 435 | -2.33 | -2.55,-2.13 | 0.10 | <0.001 |  | 148 | -2.18 | -2.54,-1.87 | 0.12 | <0.001 |
| Conifer | 79 | -1.58 | -1.97,-1.27 | 0.04 | 0.062 |  | 19 | -1.55 | -2.51,-0.96 | 0.01 | 0.422 |

*n*, sample size; *s.*CI, 95% confidence interval of slope; *r*^2^, correlation coefficient.
